# Novel Research of Dementia Prediction and Monitoring: When AI Meets Text Documents

**DOI:** 10.1093/geroni/igaf122.1203

**Published:** 2025-12-31

**Authors:** Hiroko Dodge, Liu Chen, W Quin Yow

## Abstract

Early prediction and monitoring of dementia provide a longer window for interventions, risk factor management, and future disease-modifying therapies. Various data types, such as blood samples and neuroimaging, have been explored for predicting early dementia risk, showing promising results. More recently, text documents, such as conversation transcriptions and health records, have gained attention due to their potential for AI-driven analysis and practical advantages. The accessibility, scalability, cost-effectiveness, continuous monitoring, and information-rich nature of text documents make them particularly compelling for widespread early detection. Thus, AI-driven analysis of text documents for dementia prediction deserves further investigation. This symposium highlights four novel research directions in applying AI to text documents for dementia risk prediction. Presentations will feature: 1) Quin will present an exploration evaluating the use of machine learning and AI-driven measurements for cognitive impairment detection, revealing insights into detection performance and the value of various measurement sets. 2) Hoang will present a temporal harmonization technique for longitudinal conversation, showing enhanced mild cognitive impairment (MCI) detection accuracy on data collected by I-CONECT study. 3) Jiankun will present a dementia risk prediction pipeline that combines conventional machine learning with large language models (LLMs), showing that incorporating LLM significantly improves performance. 4) Chen will present the difference in linguistic characteristics between MCI and individuals with normal cognition and MCI individuals’ changes after they participated in the I-CONECT intervention for 6 months. The symposium collectively demonstrates the promising and multifaceted potential of incorporating AI with text documents for early dementia prediction.

